# ACE2 polymorphisms impact COVID-19 severity in obese patients

**DOI:** 10.1038/s41598-022-26072-7

**Published:** 2022-12-13

**Authors:** Nour Jalaleddine, Amal Bouzid, Mahmood Hachim, Narjes Saheb Sharif-Askari, Bassam Mahboub, Abiola Senok, Rabih Halwani, Rifat A. Hamoudi, Saba Al Heialy

**Affiliations:** 1 College of Medicine, Mohammed Bin Rashid University of Medicine and Health Sciences, Dubai, UAE; 2 Sharjah Institute for Medical Research, University of Sharjah, Sharjah, UAE; 3 Department of Pulmonary Medicine and Allergy and Sleep Medicine, Rashid Hospital, Dubai Health Authority, Dubai, UAE; 4 Prince Abdullah Ben Khaled Celiac Disease Research Chair, Department of Pediatrics, Faculty of Medicine, King Saud University, Riyadh, Saudi Arabia; 5 Department of Clinical Sciences, College of Medicine, University of Sharjah, Sharjah, UAE; 6 Division of Surgery and Interventional Science, UCL, London, UK; 7 Meakins-Christie Laboratories,, Research Institute of the McGill University Health Center, Montreal, QC Canada

**Keywords:** Biochemistry, Computational biology and bioinformatics, Genetics, Immunology, Molecular biology, Biomarkers, Diseases, Medical research

## Abstract

A strong association between obesity and COVID-19 complications and a lack of prognostic factors that explain the unpredictable severity among these patients still exist despite the various vaccination programs. The expression of angiotensin converting enzyme 2 (ACE2), the main receptor for severe acute respiratory syndrome coronavirus-2 (SARS-CoV-2), is enhanced in obese individuals. The occurrence of frequent genetic single nucleotide polymorphisms (SNPs) in *ACE2* is suggested to increase COVID-19 severity. Accordingly, we hypothesize that obesity-associated *ACE2* polymorphisms increase the severity of COVID-19. In this study, we profiled eight frequently reported *ACE2* SNPs in a cohort of lean and obese COVID-19 patients (n = 82). We highlight the significant association of rs2285666, rs2048683, rs879922, and rs4240157 with increased severity in obese COVID-19 patients as compared to lean counterparts. These co-morbid-associated SNPs tend to positively correlate, hence proposing possible functional cooperation to *ACE2* regulation. In obese COVID-19 patients, rs2285666, rs879922, and rs4240157 are significantly associated with increased blood nitrogen urea and creatinine levels. In conclusion, we highlight the contribution of *ACE2* SNPs in enhancing COVID-19 severity in obese individuals. The results from this study provide a basis for further investigations required to shed light on the underlying mechanisms of COVID-19 associated SNPs in COVID-19 obese patients.

Obesity, a major epidemic and chronic disease, is a key contributing factor to adverse outcomes and hospitalization of patients with severe acute respiratory syndrome coronavirus 2 (SARS-CoV-2), the virus responsible for COVID-19. Up to 40% of the world’s population is categorized as overweight and obese, with increasing incidence by 2030^1,2^. This is alarming, as obesity has been associated with severe infections. Moreover, obese individuals are characterized by their dysregulated immune system and impaired immune responses to vaccines. This could partially explain the increased risk of mortality in COVID-19 obese patients^3,4^. Recalling the 2009 influenza pandemic, obese individuals were at higher risk of death due to the viral infection. Obese individuals were twice likely to develop influenza despite vaccination^5,6^. Taken together, this has led to extensive clinical investigations regarding COVID-19. Recent studies have highlighted the effect of obesity as a unifying risk factor for increased hospitalization and the COVID-19 mortality rate. Analysis of COVID-19 individuals with high BMI and increased waist circumference displayed an increased risk of COVID-19-related mortality^7–9^. Similarly, the increased risk of hospitalization was directly linked to increased BMI in COVID-19 subjects^9,10^. In UAE, the presence of underlying co-morbidities and high BMI work synergistically to affect the clinical outcomes of COVID-19^11^. Despite this clinical association, there is still a lack of prognostic factors that explains the variability and unpredictable severity among COVID-19 obese patients.

SARS-CoV-2 entry into the cell is dependent on Angiotensin-Converting Enzyme 2 (ACE2) receptor. ACE2 is a type I transmembrane metallocarboxypeptidase with homology to ACE, an enzyme long known to be a key player in the renin-angiotensin system (RAS), and a target for the treatment of hypertension^12,13^. The secreted protein catalyses the cleavage of the C-terminal dipeptide of Angiotensin I to produce Angiotensin 1–9 and Angiotensin II to produce Angiotensin 1–7^14^. It has been suggested that genetic variations, including single nucleotide polymorphisms (SNPs) in the *ACE2* gene, may account for the differences in symptoms and severities seen in COVID-19 patients, leading to altered immune responses and greater viral susceptibility. For example, rs182366225 and rs2097723 are two polymorphisms that may increase the expression of *ACE2* with a higher prevalence in the East Asian population. On the other hand, the rs142017934 SNP has also been associated with increased expression of *ACE2*; however, it is exclusive to Africans^15^. Residue changes in *ACE2* may potentially affect its expression or its binding affinity to the virus and raise the vulnerability of individuals to SARS-CoV-2 infection^16^. Therefore, identifying *ACE2* SNPs can shed light on the involvement of genetic variations in the epidemiological differences in COVID-19 susceptibility. Although earlier studies failed to show an association between *ACE2* polymorphisms and susceptibility or severity of COVID-19 in the general population^17^, a more recent study was able to identify four SNPs in *ACE2* which were associated with the severity of the disease. Among these, rs2106809 and rs2285666 correlated with an increased risk of hospitalization and severity of COVID-19. The presence of co-morbid conditions such as obesity increased the risk for ICU admission and death in these patients^18^.

We have previously shown that lung epithelial cells of obese patients express higher levels of *ACE2,* which may explain the increased susceptibility to infection and severe outcomes in obese patients^19^. Moreover, our group showed that obesity affects the metabolome of COVID-19 patients, where we proposed n6-acetyl-l-lysine and p-cresol as metabolic signatures having crucial roles in the poor prognosis of COVID-19 obese patients^20^. SARS-CoV-2 entry into the host cell depends on *ACE2*, thus we propose that genetic variations in *ACE2* may account for the differences in symptoms and severities seen in COVID-19 patients. To our knowledge, no studies have focused on the *ACE2* genetic variations in the COVID-19 obese population. We hypothesize that obesity is associated with genetic variants which may modulate the expression of *ACE2* and, therefore be responsible for the increased susceptibility or severity of COVID-19. This study provides insight into the correlation of frequently reported *ACE2* variants and increased severity, in association to obesity, in COVID-19 patients, which might further explain the complications seen in obese patients compared to their lean counterparts.

## Results

### Targeted Next-Generation Sequencing detected eight ACE2 polymorphisms in COVID-19 patients

**Figure 1:**
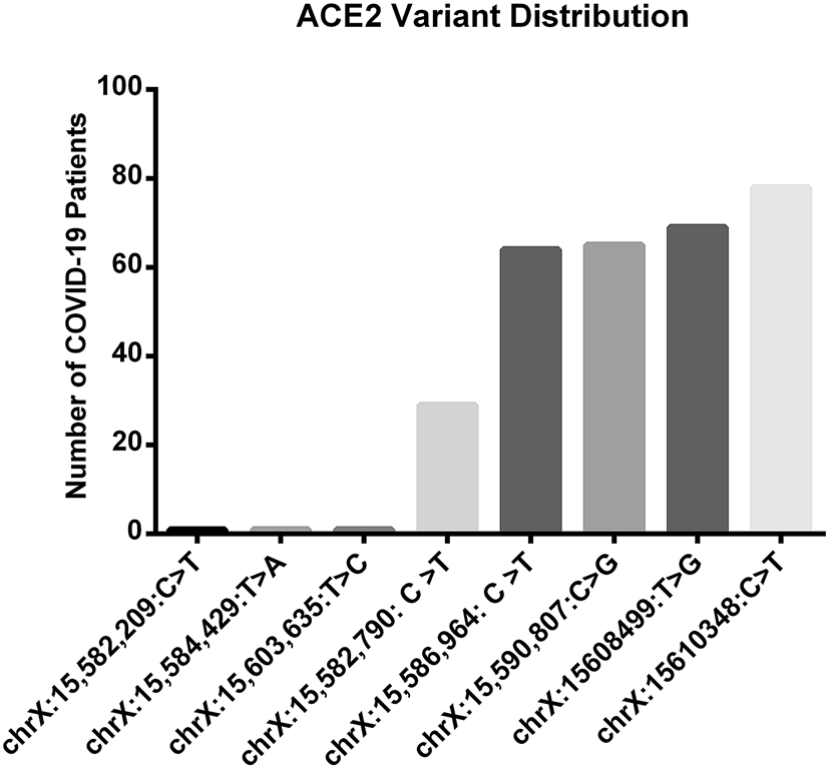
Distribution of ACE2 variants among the COVID-19 lean and obese patients. rs2285666 (chrX:15,610,348, C > T) has the highest distribution among the studied cohort; present in 79 patients; rs2048683 is present in 68 patients; rs879922 is present in 64 patients and rs4240157 is present in 61 patients.

Targeted Next-Generation Sequencing was performed in 82 COVID-19 patients for *ACE2* variants screening using the Fluidigm Access Array. Our analysis revealed 8 *ACE2* single nucleotide variants to be present among the studied population (Table 1). Interestingly, a splice variant represented as rs2285666 or as COSV53024795, at chromosome position X:g.15610348 (Fig. 1), is found to be the most frequent variant in our study cohort, being present in 78 out of the 82 COVID-19 patients (Fig. 1). Two out of the detected 8 variants, X:g.15,582,209 (rs35803318) and X:g.15,5824,429 (COSV53023851), are synonymous mutations located, respectively, at exons 18 and 17 (ENST00000427411.1; Table 1). rs35803318 was detected in only two patients that are obese and overweight, while COSV53023851 is detected in one obese patient (Fig. 1). One undetermined missense mutation (X:g.15603635) is found to be located in the coding region of *ACE2* (Table 1) and is present in only one obese patient (Fig. 1). Also, our data revealed that out of the 8 assessed variants, only 4 show potential significance in the studied population (rs2285666, rs4240157, rs879922, rs2048683). Regardless of their BMI or clinical manifestations, these mutations are present in almost up to 69 COVID-19 patients; except the rs2074192 SNP is found to be present in 29 patients, as shown in Fig. 1.

**Table 1:** *ACE2* variants (SNPs) detected in COVID-19 lean and obese patients. Eight exonic and intronic *ACE2* variants were detected in the 82 COVID-19 subjects using Targeted-NGS.

Variation	Consequence	EXON	INTRON	cDNA position	CDS position	Protein position	Amino acids	Codons	Existing variation
X:g.15582209C > T	synonymous_variant	18/19	–	2464	2247	749	V	gtG/gtA	rs35803318
X:g.15582790C > T	intron_variant	–	17/18	–	–	–	–	–	rs2074192
X:g.15584429 T > A	synonymous_variant	17/19	–	2278	2061	687	A	gcA/gcT	COSV53023851
X:g.15586964C > T	intron_variant	–	15/18	–	–	–	–	–	rs4240157
X:g.15590807C > G	intron_variant	–	12/18	–	–	–	–	–	rs879922
X:g.15603635 T > C	missense_variant	8/19	-	1080	863	288	K/R	aAa/aGa	
X:g.15608499 T > G	intron_variant	–	5/18	–	–	–	–	–	rs2048683
X:g.15610348C > T	splice_region	–	4/18	–	–	–	–	–	rs2285666, COSV53024795

### Potential association of rs2285666, rs4240157, rs879922, and rs2048683 with increased SARS-CoV-2 susceptibility and severity in obese COVID-19 patients

**Figure 2:**
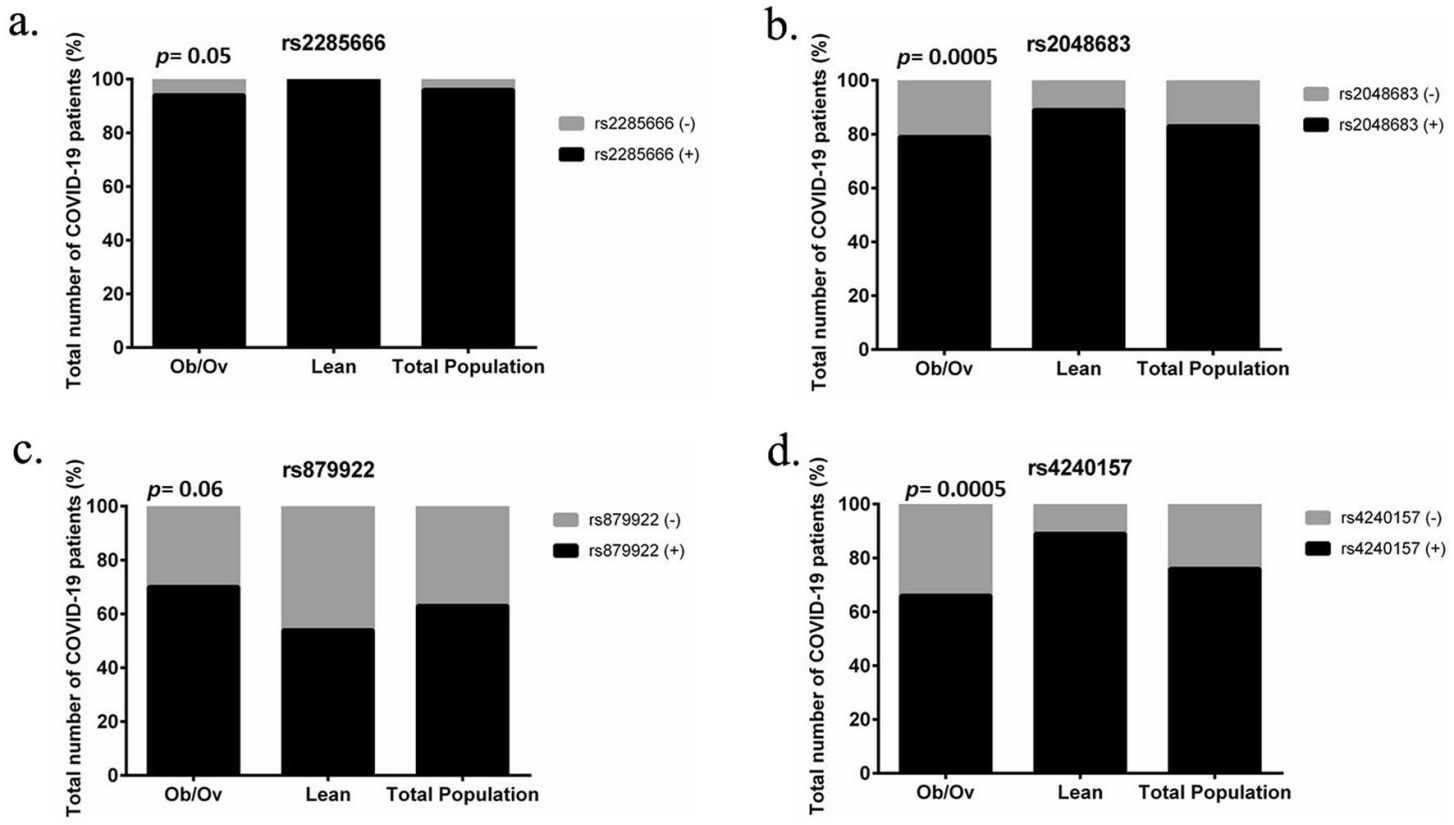
*ACE2* variants are correlated to the BMI of COVID-19 patients. (**a**) rs2285666 and (**b**) rs2048683 (**c**) rs879922 and (**d**) rs4240157 are the most significantly correlated to the body mass index (BMI) of COVID-19 lean and obese/overweight subjects. The variant (**c**) rs879922 is almost significantly expressed in the COVID-19 obese subjects (70%) more readily than in the COVID-19 lean subjects (54%). Contingency test using Chi-square analysis was applied for each variant.

**Figure 3:**
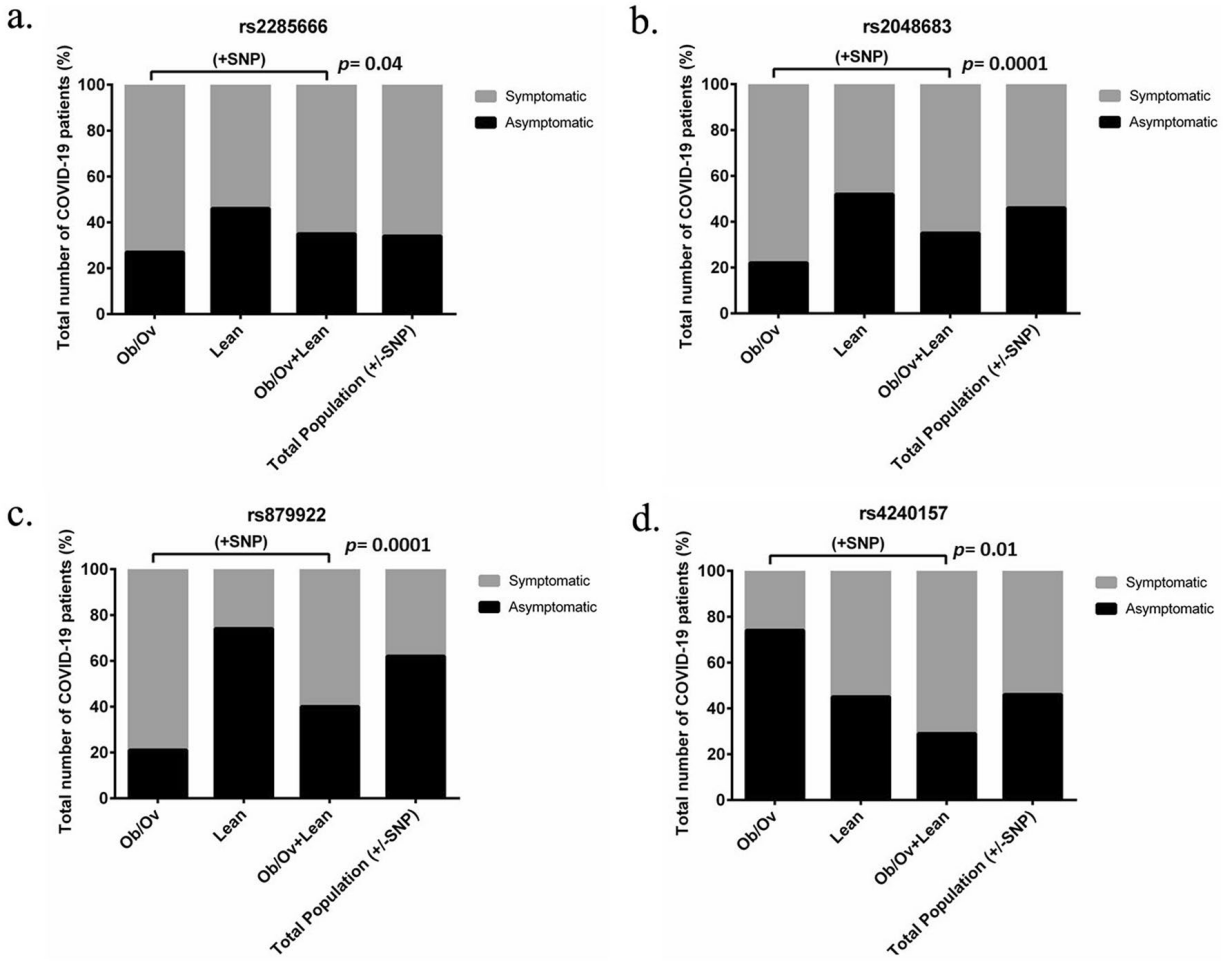
COVID-19 obese subjects expressing *ACE2* variants are more symptomatic than COVID-19 lean subjects. (**a**) rs2285666, (**b**) rs2048683, and (**c**) rs879922 are significantly correlated to the incidence of symptoms in COVID-19 obese/overweight subjects. The variant (**d**) rs4240157 is significantly correlated to the incidence of symptoms in COVID-19 lean subjects. A contingency test using Chi-square analysis was applied for each variant.

Next, we evaluated the possible involvement of the identified 8 genetic variants in the *ACE2* gene in COVID-19 susceptibility by assessing the frequency distribution of each variant among the different BMI groups (Fig. 2), including the symptoms development status, referred as asymptomatic versus symptomatic; (Fig. 3). Our data showed a significantly higher distribution of rs2285666 (*p* = 0.05), rs2048683 (*p* = 0.0005) and rs4240157 (*p* = 0.0005) in lean COVID-19 patients (Fig. 2a,b,d), while the rs879922 SNP was almost potentially significant in COVID-19 obese and overweight (Ob/Ov) patients (*p* = 0.06; Fig. 2c). Furthermore, our data showed that the SNPs rs2285666, rs2048683, and rs879922 are significantly correlated with symptoms development of COVID-19 in Ob/Ov subjects (Fig. 3a–c), with *p* = 0.04, *p* = 0.0001, and *p* = 0.0001, respectively. Only rs4240157 is mainly correlated to the incidence of symptoms in COVID-19 lean subjects (*p* = 0.01; Fig. 3d). Consequently, these SNPs could be associated with symptomatic risk and severity of COVID-19 infection. On the other hand, our analysis displayed no significant correlation between rs2074192, rs35803318, COSv53023851, and the exonic variant chrX:15,603,635, among the studied cohort and their BMI (Table 2). Similarly, no significant correlation was detected between these SNPs and the symptom phenotype of COVID-19 (Table 2). Table 2 summarizes the significance of each SNP with BMI and their potential for COVID-19 symptom development.

**Table 2:** A summary of the significance of ACE2 variants according to BMI and COVID-19 Severity in COVID-19 patients.

Variation	Existing variation	Significance to BMI (*p* value)	Significance to COVID-19 Severity (*p* value)
X:g.15582209C > T	rs35803318	0.129	0.99
X:g.15582790C > T	rs2074192	0.95	0.07
X:g.15584429 T > A	COSV53023851	0.129	0.34
X:g.15586964C > T	rs4240157	0.0005	0.01
X:g.15590807C > G	rs879922	0.06	0.0001
X:g.15603635 T > C		0.364	0.76
X:g.15608499 T > G	rs2048683	0.0005	0.0001
X:g.15610348C > T	rs2285666, COSV53024795	0.05	0.04

**Figure 4:**
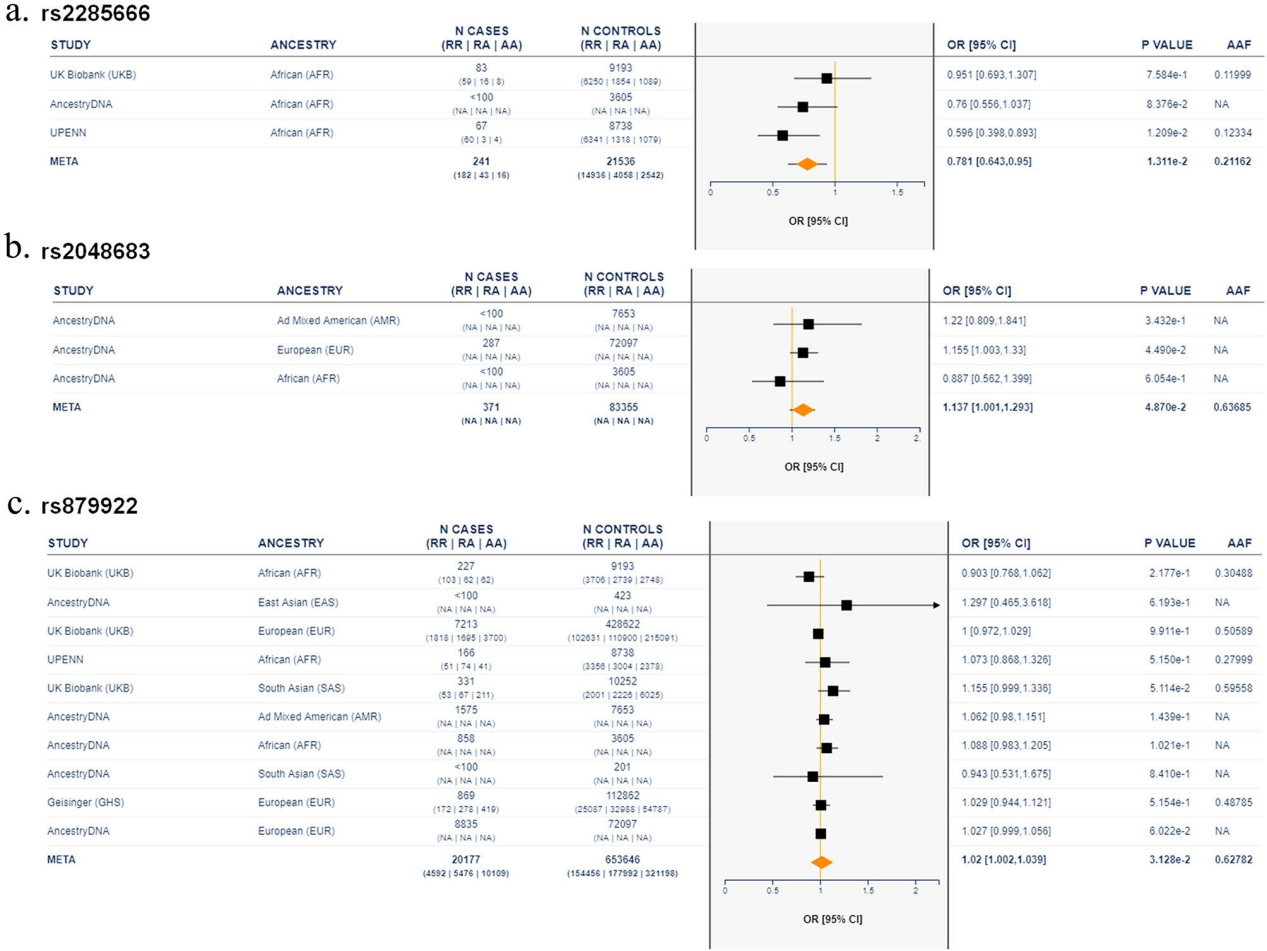
Association between COVID-19 outcomes and the most significant variants: rs2285666, rs2048683, and rs879922. Results combined across cohorts using an inverse variance meta-analysis showing Odd ratios (Confidence interval; [95% CI) for (**a**) rs2285666 (n = 241 COVID-19-positive cases, n = 21,536 COVID-19-negative), (**b**) rs2048683 (n = 371 COVID-19-positive cases, n = 83,355 COVID-19-negative), and (**c**) rs879922(n = 20,177 COVID-19-positive cases, n = 653,646 COVID-19-negative). RR, individuals who have genotype reference/reference for all variants included in burden test; RA, individuals who have genotype reference/alternate for at least one variant; AA, individuals who have genotype alternate/alternate for at least one variant; AAF, alternative allele frequency.

Given the potential significance of these findings, we then examined the frequency of the significantly correlated SNPs in our cohort by addressing the most extensive database for the COVID-19 associated variants, “A catalogue of associations between rare coding variants and COVID-19 outcomes” ^21,22^, using the online platform (https://rgc-covid19.regeneron.com/). Tables provided as supplementary (Tables S1, S2, and S3) show the list of studies with a significant association of each of the detected SNPs from our study in patients with COVID-19. Figure 4 shows the meta-analysis Odds ratio between different studies that included the alternative allele frequency (AAF) for these SNPs: rs2285666 (AAF: 021,162); rs2048683 (AAF: 0.63685); rs879922 (AAF: 0.62782). Interestingly, the African ancestry was one of the populations where the effect of rs2285666, rs2048683, and rs879922 are shown to direct towards a significant detrimental effect (Fig. 4a–c) when compared to other population groups. This implies that these SNPs might have a different impact based on different ethnicities.

### ACE2 variants increase blood biomarkers in COVID-19 patients

**Figure 5:**
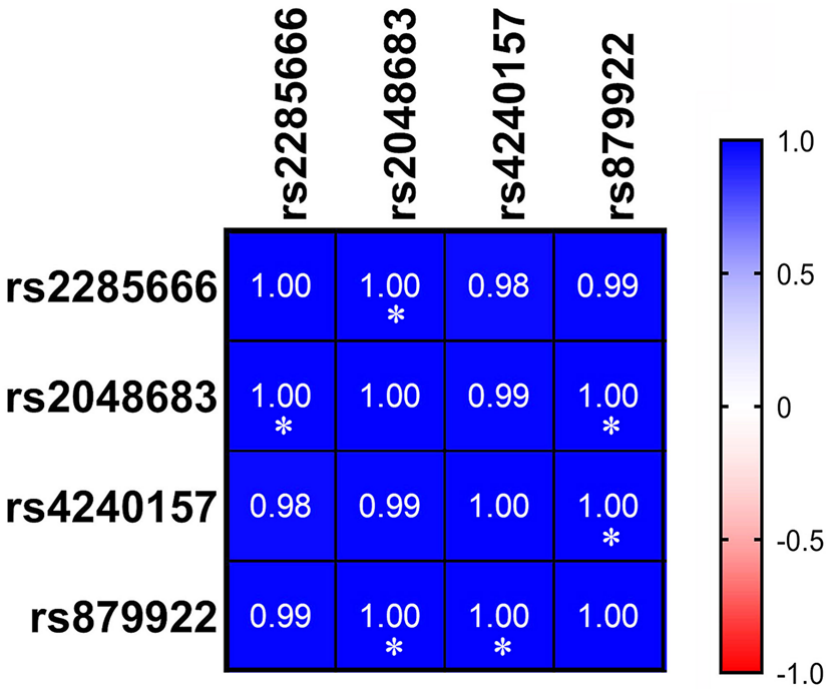
Positive correlation between *ACE2* variants. Correlation plot showing the correlation between the different detected SNPs. The heatmap colors reflect rs2285666, rs2048683, rs4240157, and rs879922 are the most significantly correlated. Pearson correlation coefficients with blue for positive correlation and red for negative correlation. *p < 0.05; **p < 0.01.

**Figure 6:**
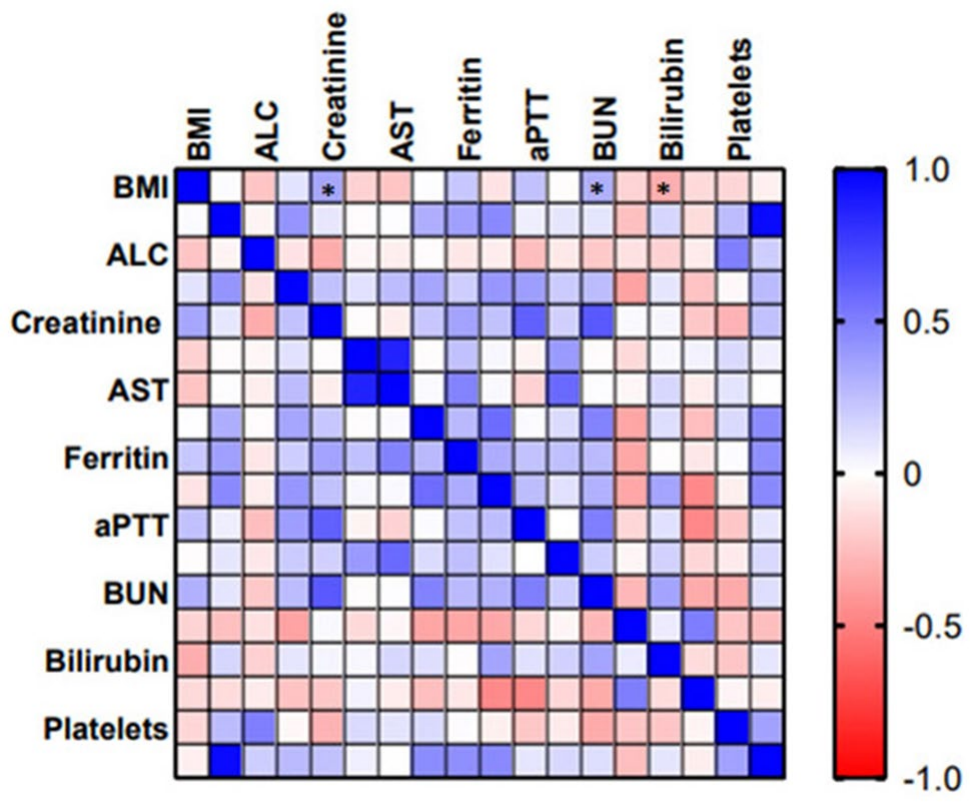
Correlation representation between BMI and blood biomarkers. A correlation plot showing that the BMI of the studied cohort and their clinical blood biomarkers. Pearson correlation coefficients with blue for positive correlation and red for negative correlation. *p < 0.05.

To better understand the relationship between the identified SNPs, the BMI, and the severity of symptoms due to COVID-19, a Pearson correlation analysis was performed between the occurrence of the identified SNPs and the number of patients with COVID-19. A graphical representation of the association is shown in Fig. 5. All identified SNPs were positively correlated, irrespective of the severity and BMI. For instance, rs2048683 is significantly correlated with rs879922 (*p* = 0.03) and with rs2285666 (*p* = 0.04), and rs4240157 is almost significantly correlated with rs879922 (*p* = 0.05), as shown in Fig. 5. These identified correlated SNPs are known to affect *ACE2*-SARS-CoV-2 binding affinity and COVID-19 severity^23,24^. Moreover, correlation analysis has been performed between the BMI of the studied cohort expressing these SNPs and their clinical information. Our analysis showed that BMI correlates significantly with blood urea nitrogen (BUN) and creatinine blood levels (Fig. 6), implying kidney complications or failure.

**Figure 7:**
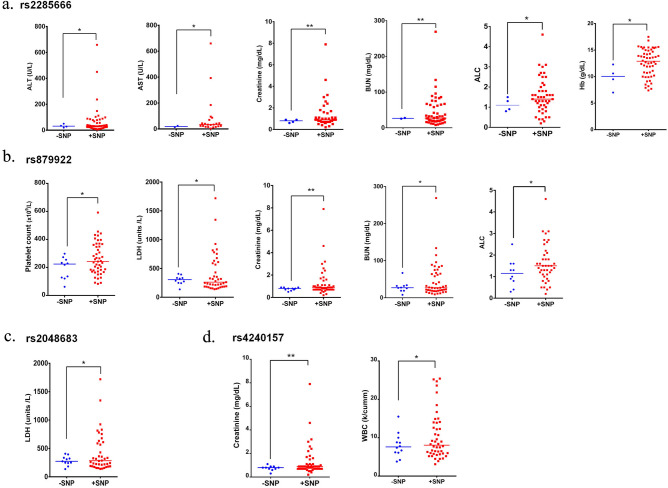
Significant association between rs228566, rs2048683, rs4240157 and rs879922 and elevated levels of blood biomarkers. Significant increase in the levels of ALT, AST, Hb, ALC, creatinine, and BUN clinical tests in COVID-19 patients expressing (**a**) rs2285666. In COVID-19 patients expressing (**b**) rs879922 revealed significant increase in the levels of ALC, creatinine, BUN, and LDH, in addition to platelets count. Increased levels in creatinine, LDH and WBC count in COVID-19 patients with (**c**) rs2048683 and (**d**) rs4240157. *p < 0.05; **p < 0.01. *ALT* alanine aminotransferase; *AST* aspartate aminotransferase; *Hb* *hemoglobin*; *ALC* absolute lymphocyte count; *BUN* blood urea nitrogen; *LDH* lactate dehydrogenase; *WBC* white blood cells.

A further analysis was performed to understand the exact correlation between rs2285666, rs4240157, rs879922, and rs2048683 and the clinical variability among the COVID-19 patients. Interestingly, our data showed that each of rs2285666, rs4240157, rs879922, and rs2048683 are significantly associated with increased levels of either of alanine aminotransferase (ALT), aspartate aminotransferase (AST), *haemoglobin* (Hb), absolute lymphocyte count (ALC), lactate dehydrogenase (LDH), BUN and creatinine, in addition to white blood cells (WBC) and platelets count (Fig. 7). COVID-19 patients expressing rs2285666 mainly display significant increase in ALT (*p* = 0.0315), AST(*p* = 0.0261), Hb (*p* = 0.02), ALC (*p* = 0.039), creatinine (*p* = 0.008), and BUN (*p* = 0.0027) blood levels (Fig. 7a). COVID-19 patients expressing rs879922 revealed significant increase in ALC (*p* = 0.048), creatinine (*p* = 0.0041), BUN (*p* = 0.0215), and LDH (*p* = 0.0026) blood levels, in addition to platelets count (*p* = 0.018; Fig. 7b). COVID-19 patients expressing rs2048683 and rs4240157 displayed increase in LDH (*p* = 0.0112) and creatinine (*p* = 0.005) blood levels, and in WBC count (*p* = 0.0444; Fig. 7c,d), respectively.

**Figure 8:**
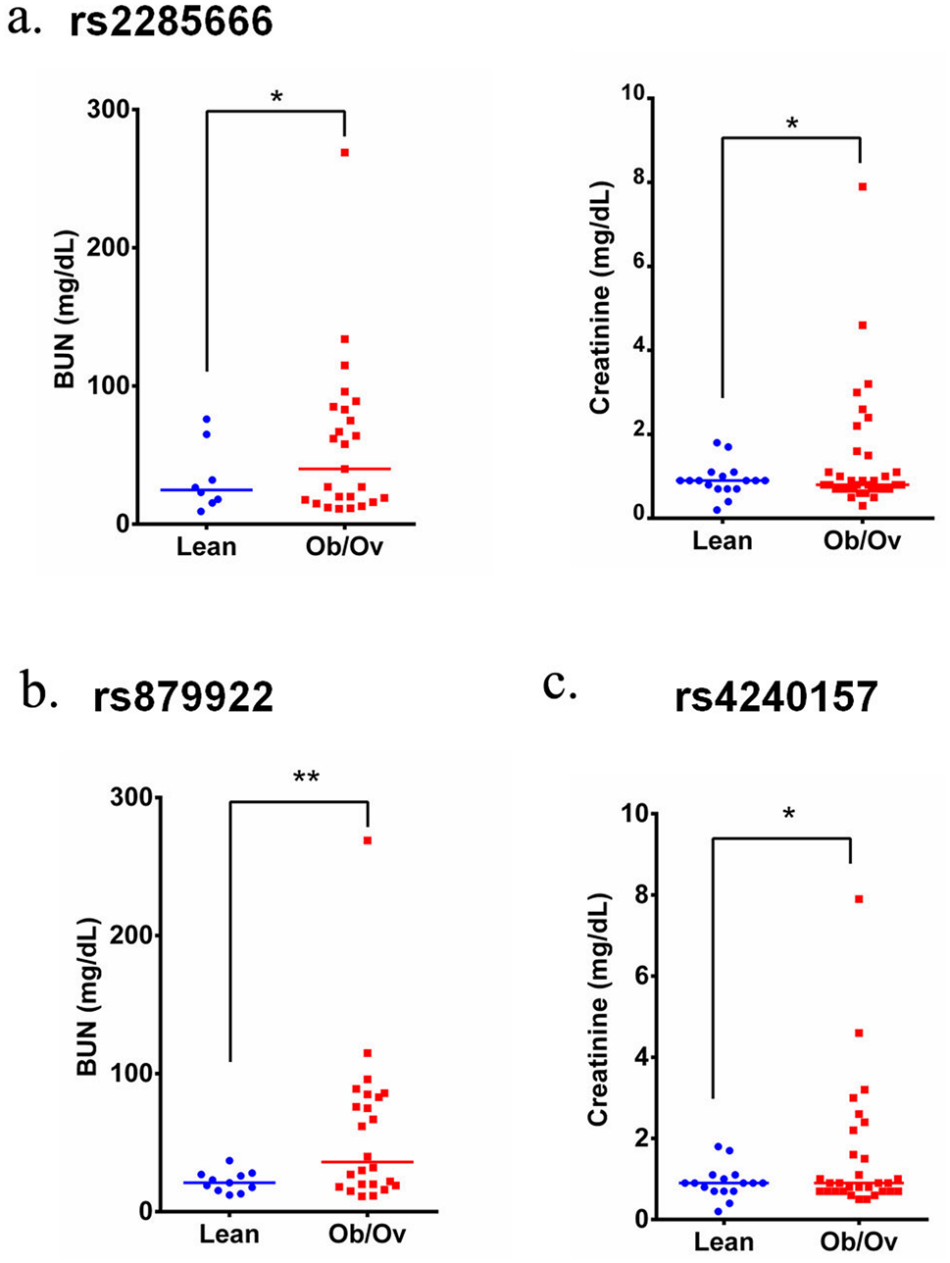
Significant association between *ACE2* variants and blood biomarkers in COVID-19 obese patients. Creatinine and BUN levels are significantly increased in COVID-19 patients obese patients expressing (**a**) rs2285666, (**b**) rs879922, and (**c**) rs4240157 variants as compared to their lean counterparts. *p < 0.05; **p < 0.01.

This analysis was further stratified into assessing the correlation of these SNPs with BMI in the studied cohort. Interestingly, COVID-19 Ob/Ov patients expressing rs2285666, rs879922, and rs4240157 displayed significant increase in creatinine (*p* = 0.0394 and *p* = 0.0292) and BUN blood levels (*p* = 0.047 and *p* = 0.0023; Fig. 8a–c), respectively. This comes in parallel with our correlation analysis in Fig. 6, which further indicates the potential association of these SNPs with kidney health.

Taken together, these data point out that the association of these *ACE2* SNPs with COVID-19 severity is also in part related to obesity and possibly obesity-associated co-morbidities in these patients.

## Discussion

Many studies have shown a positive association between obesity and COVID-19 severity. However, and to our knowledge, this is the first study to evaluate the contribution of obesity in SARS-CoV-2 infection susceptibility that may be associated with certain genomic variants within the *ACE2* gene and thus might modulate its expression and function. We report the profiling and possible involvement of four out of eight detected *ACE2* single nucleotide polymorphisms (SNPs; rs2285666, rs2048683, rs879922, and rs4240157) in offering a plausible biological explanation for the increased expression of *ACE2* and health complications seen in obese COVID-19 subjects.

Polymorphisms of more than twenty obesity-related genes have been documented to correlate with metabolic dysregulation and low-grade inflammation in different diseases, including viral infections^25,26^. Moreover, it was suggested that these metabolically related polymorphisms might interfere with SARS-CoV-2 intrusions and antiviral responses^27^. Considering this association between gene polymorphisms, obesity and SARS-CoV-2 infection, it is important to decipher the effect of obesity on *ACE2* polymorphisms, as being the primary entry site for SARS-CoV-2, with evidence of higher expression in obese individuals^19,28^. In this study, we have identified eight *ACE2* genetic variations, among which rs2285666, rs2048683, rs879922, and rs4240157 are the most significantly present in the studied cohort that comprises lean and obese COVID-19 subjects. These SNPs were found to be commonly distributed in both lean and obese COVID-19 subjects. On the other hand, our analysis has shown that overweight and obese COVID-19 subjects who have these SNPs tend to be significantly more symptomatic as compared to lean COVID-19 subjects. Importantly, the rs879922 SNP was significantly correlated with both BMI and severity in COVID-19 obese and overweight subjects as compared to COVID-19 lean subjects. Among the detected *ACE2* variants, the rs2285666 SNP is the most distributed polymorphism in the studied population. Several studies have highlighted a genetic association of *ACE2* rs2285666 polymorphism with elevated *ACE2* gene expression in different populations that involves Europeans, Asians, and Indians. The upregulated *ACE2* expression is reported to be up to 50%, despite the striking difference in allele frequency in the different populations^29–32^ , hence suggesting a positive association between rs2285666 and increased susceptibility to COVID-19. Of note, rs2285666 is considered one of the variants that enhances the binding affinity of SARS-CoV-2 to ACE2, enhancing its infection, and thus impacting severe clinical outcomes related to SARS-CoV-2 infection^23,33,34^. On the other hand, a few other reports also suggested a positive correlation between the SNPs rs4240157, rs204683 and rs879922 with higher tissue expression of *ACE2* and correlated it with increased hospitalisations of COVID-19 patients^24^. This aligns with our data, where our analysis showed that these SNPs, rs2285666, rs4240157, rs204683 and rs879922, positively correlate with each other, thus indicating possible functional cooperation and contribution to *ACE2* regulation. In addition, our data showed a significant and positive association between the BMI of these patients and health complications, including kidney and liver malfunctions and associated inflammation due to increased WBC and ALC levels. Interestingly, clinical information of COVID-19 obese subjects expressing *ACE2* rs2285666, rs879922, and rs4240157 variants reflected increased kidney complications due to the significant increase of creatinine and BUN blood levels. The rs2285666, rs2048683, rs879922, and rs4240157 variants are known to be associated with type 2 diabetes mellitus (T2DM) and obesity related co-morbidities such as cardiovascular disease and hypertension^30,32,35^. Overall, these data suggest the coordination of these SNPs in contributing to *ACE2* expression that may possibly be a predisposing factor associated with the co-morbidities observed in COVID-19 patients, especially in obese subjects.

**Figure 9:**
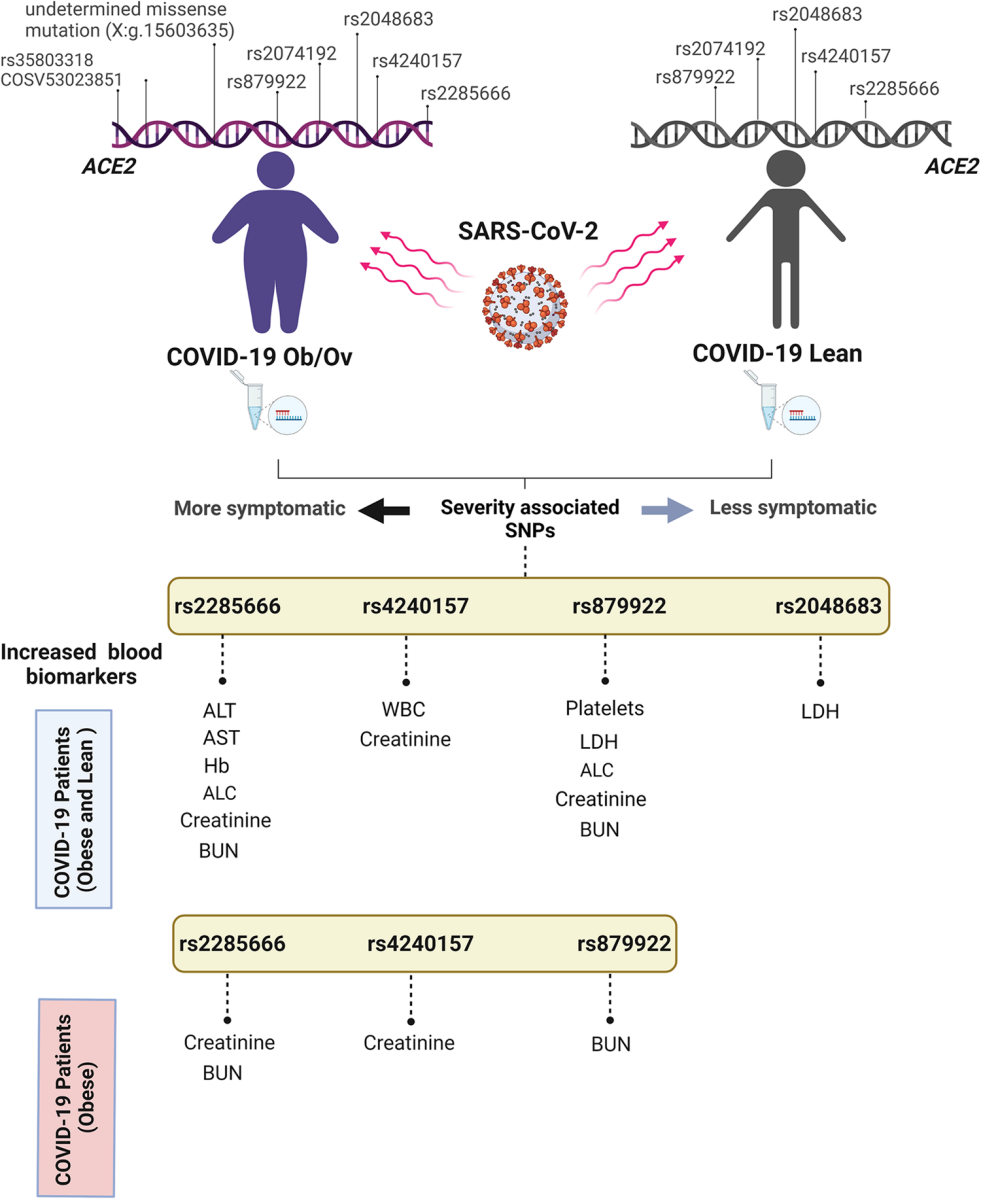
A representative scheme summarizing the *ACE2* variants impact on blood biomarkers in COVID-19 lean and obese patients.

In summary, this study lays the foundation for future studies to bridge the gap between the polymorphisms and their possible involvement in the dysregulation of *ACE2* in COVID-19. A limitation to our study includes the lack control samples (non-COVID-19) due to the difficulty in achieving patients’ samples that were not vaccinated, as vaccination process in the United Arab Emirates (UAE) has been initiated effectively by treated all people as emergency patients^36,37^. Moreover, additional information regarding demographic and pre-existing conditions such as smoking status for each patient were not provided. Despite the low number of non-vaccinated COVID-19 patients’ samples that we obtained, our data has highlighted the correlation of the frequently reported ACE2 SNPs (rs2285666, rs2048683, rs879922, and rs4240157) and symptoms development of COVID-19 patients. Unifying our understanding of obesity with severe health complications in COVID-19, we propose an underlying health complication that correlates *ACE2* SNPs with poor COVID-19 outcomes (Fig. 9). We highlight the importance of *ACE2* genetic polymorphisms, as it may be an effective method to enhance the efficacy of immunotherapeutic and conventional treatments in COVID-19 patients and specifically in obese COVID-19 patients.

## Materials and methods

### Patients and sample processing

Fasting blood samples were collected from a cohort of COVID-19 patients (n = 82) who visited Rashid Hospital (Dubai, UAE) between June and July 2020. Blood samples were then recruited from the COVID-19 Biobank for further analysis through a collaboration with the University of Sharjah (Sharjah, UAE). The studied population was categorized according to their body mass index (BMI) as follows; Patients with BMI ≥ 30 kg/m^2^, BMI = 25–29.9 kg/m^2^, and BMI < 25 kg/m^2^ are classified as obese (n = 28), overweight (n = 19), and lean (n = 35), respectively. Table 3 describes patients’ characteristics. Vaccinated COVID-19 samples have been excluded from the selection criteria. Patients’ COVID-19 clinical manifestations ranged from severe to moderate and mild symptoms and asymptomatic status.

**Table 3:** COVID-19 patients’ characteristics.

COVID-19 subjects characteristics		
Parameters	Lean	Obese/overweight
Subjects (N)	35	47
Age (years)	42 ± 15	50 ± 16
Gender, M/ F	22/13	38/9
BMI (kg/m^2^)	22 ± 2	30 ± 4
**Serum markers**		
AST (U/L)	28.5 (393–3.2)	31 (660–12)
ALT (U/L)	33 (660–3.2)	32 (659–6)
Hb (g/dL)	13.25 (15.7–7)	12.9 (16.8–7)
ALC	1.4 (3.1–0.2)	1.4 (4.6–0.3)
Creatinine (mg/dL)	0.9 (7.9–0.2)	0.8 (2.6–0.4)
Ferritin (µg/L)	505 (5147–5)	669 (6661–38)
aPTT (sec)	38.5 (63.2–31.8)	40.25 (64.7–31.9)
BUN (mg/dL)	28 (134–9.3)	27.5 (269–8)
WBC (k/cumm)	7.6 (16.7–3.1)	8 (25.4–4.2)
Platelets (× 10^3^/ L)	219 (456–62)	237.5 (592–91)
LDH (units/L)	268 (925–137)	283 (1719–140)
Bilirubin (µmol/L)	0.7 (48.2–0.2)	0.7 (11.2–0.2)
**Medication (%)**		
Metformin, Diamicron, Statins, Insulin	5%	46%
Plavix, Aspirin	3%	11%
**Nationality (%) (Geographical zones)**		
Middle East	3%	9%
Eastern and Southern Asia	35%	49%
Northern and Southern America	1%	2%
Africa	1%	–

*BMI* body mass index; *AST* aspartate aminotransferase test, *ALT* alanine aminotransferase test; *Hb* haemoglobin; *ALC* haemoglobin A1c test; *aPTT* activated partial thromboplastin clotting time; *BUN* blood urea nitrogen; *WBC* white blood cell; *LDH* lactate dehydrogenase test.

Values are shown as mean ± SD; median ( confidence intervals); %, percentage.

Briefly, 8 ml of peripheral venous blood were collected in Ethylenediamine Tetra Acetic Acid (EDTA)- blood collection tubes. Within 24 h, collected blood samples were processed using density gradient media by diluting them with Phosphate Buffered Saline (PBS (1x); Sigma-Aldrich, USA) containing 2% human fetal bovine serum (FBS; Sigma-Aldrich, USA), in a 1:1 ratio, mixed thoroughly, and layered onto a HISTOPAQUE®-1077gradient media (Sigma-Aldrich, USA). Several centrifugations were performed at 400 × g for 30 min at room temperature to separate blood components. Blood clots were collected and stored at − 80 °C until DNA extraction.

All patients provided written informed consent. Ethical approval was obtained from the Dubai Scientific Research Ethics committee (DSREC-04/2020_19) of the Dubai Health Authority (DHA) in the United Arab Emirates (UAE).

### DNA extraction

Genomic DNA was extracted using DNA Mini Kit -QIAMP (QIAGEN, Germany). Briefly, 20 µl of Proteinase K was added to a 200 µl of blood clot sample in an Eppendorf. Lysis buffer was then added to the mixture, where it was allowed for 10 min incubation at 70 °C. A volume of 200 µl of ethanol (96–100%) was then added to minimize DNA solubility, and the mixture was then transferred into a DNeasy Mini spin column, where several centrifugation steps were performed at room temperature; as per the manufacturer’s protocol. Eventually, DNA was eluted in 30 µl of Nuclease Free water (Invitrogen, UK). Using Nano-drop 8000 (Thermo-Scientific, USA), the quantity of extracted DNA from 200 μl of blood clot samples ranged from 24.5 ng/μl to 199.3 ng/μl, and the purity (OD260/OD280) range was 1.8–1.9; for all samples.

### ACE2 primers designing and validation

Primers were designed to cover all exonic and some intronic regions of the *ACE2* gene (Gene ID: 59,272, NM_001371415.1). The details of the *ACE2* primers are listed in Supplementary Table (S4). The primers were then assessed using control DNA samples, and the expected amplicon sizes were confirmed using agarose gel electrophoresis.

### Targeted next-generation sequencing (NGS)

The library for DNA sequencing using NGS was generated using the Fluidigm Access Array microfluidic chip as previously described^38^. The confirmed target-specific ACE2 primers were then tagged with Fluidigm specific tag sequences and used for targeted next-generation sequencing^38^. The prepared amplicon libraries were purified using AMPure XP beads (Beckman Coulter, USA) and quantified using a High Sensitivity DNA assay kit on BioAnalyzer (Agilent, USA). The libraries were further diluted to 1 pg for direct input into the emulsion polymerase chain reaction (PCR) with Ion SphereTM particles using the Ion Template OT2 kit (Ion OneTouch™ instrument). Enrichment of the clonal beads was carried out using the Ion OneTouch™ ES system following the manufacturer’s instructions (Thermo Fischer, USA). The pooled libraries were then sequenced using the Ion 520™ Chip on the Ion S5 XL Semiconductor sequencer, following the manufacturer’s instructions (Thermo Fisher).

### Data processing and targeted genetic analysis

Reads were aligned to the reference Human genome (NCBI37/hg19) by burrows wheeler aligner (BWA) on default settings^39^. Unmapped reads were excluded from the analysis. Mapped reads were visualized using an integrative genomics viewer (IGV) with the relevant browser extensible data (BED) file for the *ACE2* gene. Reads were analyzed using an in-house bioinformatics pipeline that incorporates samtools mpileup. Post-processing quality filters were applied to the output to improve specificity of downstream analysis. Resulting variants were annotated according to the Ensemble release 105 (Dec 2021). Exonic variants were further tested for pathogenicity using the *in-silico* tools PolyPhen2^40^, SIFT^41^ , and Mutation Taster^42^.

### Pan-ancestry exome-wide association analyses of COVID-19 outcomes

Exome-wide association analyses for the identified SNPs from our cohort were done using a publicly available database using the “A catalogue of associations between rare coding variants and COVID-19 outcomes”^21,22^, retrieved from the online platform (https://rgc-covid19.regeneron.com/). This catalogue was generated to identify rare variants (RVs, minor allele frequency [MAF]. < 1%) associated with COVID-19 susceptibility and severity, an exome-wide sequencing data was generated for 543,213 individuals from different studies (Geisinger Health System [GHS], Penn Medicine BioBank [PMBB] and UK Biobank [UKB]) and three ancestries (African, European and South Asian). The same phenotypes obtained from these studies were used to validate the association with common risk variants reported in our study, thus demonstrating that our phenotypes are calibrated with those used in other studies.

### Statistical analysis

Microsoft Excel and GraphPad Prism software were used to perform statistical analysis. Results are expressed as individual data or as mean ± standard deviation. Regarding COVID-19 patients, and prior to analysis, data were tested for normality using Shapiro Wilk’s normality tests. Frequencies (number of mutated patients) and percentages were used as appropriate. Data were analyzed via the Chi-square test. An unpaired *t*-test with Welch’s correction was used to compare clinical data among the groups of COVID-19 patients. A correlation plot was generated using Pearson correlation test. Statistical significance was accepted at *p* < 0.05.

### Ethical approval

The study was conducted in accordance with the Declaration of Helsinki, and ethical approval was obtained from Dubai Scientific Research Ethics committee (DSREC-04/2020_19) of Dubai Health Authority (DHA) in the United Arab Emirates (UAE).

### Informed consent

Informed consent was obtained from all subjects involved in the study.

## Supplementary Information

Supplementary Information 1.

## Data Availability

All data are contained within the manuscript.
