# Supplementary material for: ACE2 polymorphisms impact COVID-19 severity in obese patients
Source: Sci Rep. 2022 Dec 13;12:21491. doi: 10.1038/s41598-022-26072-7 (PMC9748040; doi:10.1038/s41598-022-26072-7)
Supplement: Supplementary file 1 — Supplementary Information 1. [file 41598_2022_26072_MOESM1_ESM.docx]

**ACE2 Polymorphisms Impact COVID-19 Severity in Obese Patients**

Nour Jalaleddine ^1^, Amal Bouzid ^2^, Mahmood Hachim ^1^, Narjes Saheb Sharif-Askari ^2^, Bassam Mahboub ^2,3^, Abiola Senok ^1^, Rabih Halwani ^2,4,5^, Rifat A. Hamoudi ^2,6^, Saba Al Heialy ^1,7*^

^1^ College of Medicine, Mohammed Bin Rashid University of Medicine and Health Sciences, Dubai, United Arab Emirates.

^2^ Sharjah Institute for Medical Research, University of Sharjah, Sharjah, United Arab Emirates.

^3^ Department of Pulmonary Medicine and Allergy and Sleep Medicine, Rashid Hospital, Dubai Health Authority, Dubai, United Arab Emirates.

^4^ Prince Abdullah Ben Khaled Celiac Disease Research Chair, Department of Pediatrics, Faculty of Medicine, King Saud University, Riyadh, Saudi Arabia.

^5^ Department of Clinical Sciences, College of Medicine, University of Sharjah, Sharjah, United Arab Emirates.

^6^ Division of Surgery and Interventional Science, UCL, London, United Kingdom.

^7^ Meakins-Christie Laboratories, Research Institute of the McGill University Health Center, Montreal, QC, Canada.

*****Correspondence: Saba Al Heialy; Email: [Saba.Alheialy@mbru.ac.ae](mailto:Saba.Alheialy@mbru.ac.ae) , Tel: +971-4383[**8720**](tel:8720).

**Abstract:** Despite the strong association between obesity and COVID-19 complications, there is still a lack of prognostic factors that explain the unpredictable severity among these patients. The occurrence of frequent genetic single nucleotide polymorphisms (SNPs) in the angiotensin converting enzyme 2 (ACE2), the main receptor for severe acute respiratory syndrome coronavirus-2 (SARS-CoV-2), is suggested to increase COVID-19 severity. Accordingly, we hypothesize that obesity-associated *ACE2* polymorphisms increase the severity of COVID-19 disease. In this study, we profiled eight frequently reported *ACE2* SNPs in a cohort of lean and obese COVID-19 patients (n=82). We highlight the significant association of rs2285666, rs2048683, rs879922, and rs4240157 with increased severity in obese COVID-19 patients as compared to lean counterparts. These co-morbid-associated SNPs tend to positively correlate, hence proposing possible functional cooperation to *ACE2* regulation. In obese COVID-19 patients, rs2285666, rs879922, and rs4240157 are significantly associated with increased blood nitrogen urea and creatinine levels. In conclusion, we highlight the contribution of *ACE2* SNPs in enhancing COVID-19 severity in obese individuals. The results from this study provide a basis for further investigations required to shed light on the underlying mechanisms of COVID-19 associated SNPs in COVID-19 obese patients.

**Keywords:** Obesity, ACE2, SNPs, COVID-19, SARS-CoV-2

**Supplementary Tables:**

**Table S1: rs879922 significantly associated with list of studies extracted from the online platform (**[**https://rgc-covid19.regeneron.com/**](https://eur01.safelinks.protection.outlook.com/?url=https%3A%2F%2Frgc-covid19.regeneron.com%2F&data=05%7C01%7CNour.jalaleddine%40mbru.ac.ae%7Ce4fc00eecfb249f216dc08da22a76882%7Cc9a341c74b6f4e56b8d9f2325c282fda%7C0%7C0%7C637860399660061885%7CUnknown%7CTWFpbGZsb3d8eyJWIjoiMC4wLjAwMDAiLCJQIjoiV2luMzIiLCJBTiI6Ik1haWwiLCJXVCI6Mn0%3D%7C3000%7C%7C%7C&sdata=MfqVOOee7XZek9NIm2NHvHJevx8sp4eVR0H9d6X6JN0%3D&reserved=0)**)**

| **Gene** | **Variant** | **Variant type / HGVS** | **Analysis** | **Phenotype** | **Study** | **Genetic dataset** | **Ancestry** | **Effect (OR) [CI]** | **P Value** | **Case Counts / Controls (RR:RA:AA)** | **AAF** |
| --- | --- | --- | --- | --- | --- | --- | --- | --- | --- | --- | --- |
| **N/A** | 23:15572684:C:G | intergenic | Meta-Analysis | COVID-19 positive vs COVID-19 negative or COVID-19 status unknown | ANCESTRY | Imputed | Pan-ancestry | UP 1.03 [1.01, 1.06] | 9.45E-03 | Cases: -3  (-1:-1:-1) Controls: -3  (-1:-1:-1) MAC:69575 | 0.63516 |
| **N/A** | 23:15572684:C:G | intergenic | Single-Cohort | COVID-19 positive vs COVID-19 negative | ANCESTRY | Imputed | African (AFR) | UP 1.16 [1.03, 1.3] | 1.33E-02 | Cases: -3  (-1:-1:-1) Controls: -3  (-1:-1:-1) MAC:2063 | 0.492799 |
| **N/A** | 23:15572684:C:G | intergenic | Meta-Analysis | COVID-19 positive not hospitalized vs COVID-19 negative or COVID-19 status unknown | ANCESTRY | Imputed | Pan-ancestry | UP 1.03 [1, 1.06] | 2.62E-02 | Cases: -3  (-1:-1:-1) Controls: -3  (-1:-1:-1) MAC:68969 | 0.635116 |
| **ACE2** | 23:15572684:C:G | intronic | Meta-Analysis | COVID-19 positive vs COVID-19 negative or COVID-19 status unknown | ANCESTRY, GHS, UKB, UPENN | Imputed | Pan-ancestry | UP 1.02 [1, 1.04] | 3.13E-02 | Cases:  20177  (4592:5476:10109) Controls: 653646 (154456:177992:321198) MAC:501564 | 0.627822 |

**Table S2: rs2285666 significantly associates with the list of studies extracted from the online platform (**[**https://rgc-covid19.regeneron.com/**](https://eur01.safelinks.protection.outlook.com/?url=https%3A%2F%2Frgc-covid19.regeneron.com%2F&data=05%7C01%7CNour.jalaleddine%40mbru.ac.ae%7Ce4fc00eecfb249f216dc08da22a76882%7Cc9a341c74b6f4e56b8d9f2325c282fda%7C0%7C0%7C637860399660061885%7CUnknown%7CTWFpbGZsb3d8eyJWIjoiMC4wLjAwMDAiLCJQIjoiV2luMzIiLCJBTiI6Ik1haWwiLCJXVCI6Mn0%3D%7C3000%7C%7C%7C&sdata=MfqVOOee7XZek9NIm2NHvHJevx8sp4eVR0H9d6X6JN0%3D&reserved=0)**)**

| **Gene** | **Variant** | **Variant type / HGVS** | **Analysis** | **Phenotype** | **Study** | **Genetic dataset** | **Ancestry** | **Effect (OR) [CI]** | **P Value** | **Case Counts / Controls**  **(RR:RA:AA)** | **AAF** |
| --- | --- | --- | --- | --- | --- | --- | --- | --- | --- | --- | --- |
| **ACE2** | 23:15592225:  C:T | splice_region | Single-Cohort | COVID-19 positive hospitalized vs COVID-19 negative or COVID-19 status unknown | UPENN | Imputed | African (AFR) | DOWN 0.596 [0.398, 0.893] | 0.01209 | Cases: 67  (60:3:4) Controls: 8738 (6341:1318:1079) MAC:  3487 | 0.200663 |
| **ACE2** | 23:15592225:  C:T | splice_region | Meta-Analysis | COVID-19 positive hospitalized vs COVID-19 negative or COVID-19 status unknown | ANCESTRY, UKB, UPENN | Imputed | African (AFR) | DOWN 0.781 [0.643, 0.95] | 0.01311 | Cases: 241  (182:43:  16)  Controls: 21536 (14936:  4058:  2542) MAC:  9217 | 0.211622354 |
| **ACE2** | 23:15592225:  C:T | splice_region | Single-Cohort | COVID-19 positive vs COVID-19 negative or COVID-19 status unknown | UKB | Imputed | European (EUR) | DOWN 0.957 [0.925, 0.991] | 0.01444 | Cases: 7213  (5327:1110:776) Controls: 428622 (308640:74043:  45939) MAC:  168583 | 0.192877 |
| **ACE2** | 23:15592225:  C:T | splice_region | Meta-Analysis | COVID-19 positive vs COVID-19 negative | ANCESTRY, UKB, UPENN | Imputed | African (AFR) | DOWN 0.878 [0.788, 0.978] | 0.01811 | Cases: 1251  (866:250:  135)  Controls: 3017 (2034:  640:  343) MAC:  1846 | 0.216260544 |
| **ACE2** | 23:15592225:  C:T | splice_region | Single-Cohort | COVID-19 positive vs COVID-19 negative | UKB | Imputed | European (EUR) | DOWN 0.955 [0.918, 0.994] | 0.02266 | Cases: 7213 (5327:  1110:  776) Controls: 33539 (24178:  5638:  3723) MAC:  15746 | 0.192581 |
| **ACE2** | 23:15592225:  C:T | splice_region | Single-Cohort | COVID-19 positive vs COVID-19 negative | UPENN | Imputed | African (AFR) | DOWN 0.752 [0.575, 0.982] | 0.03667 | Cases: 166 (132:18:  16) Controls: 959 (678:156:125) MAC:456 | 0.20566 |
| **ACE2** | 23:15592225:  C:T | splice_region | Meta-Analysis | COVID-19 positive vs COVID-19 negative or COVID-19 status unknown | UKB | Imputed | Pan-ancestry | DOWN 0.966 [0.934, 0.999] | 0.04187 | Cases: 7771(5627:1229:915) Controls: 448067 (319590:78303:  50174) MAC:  181710 | 0.19931423 |
| **ACE2** | 23:15592225:  C:T | splice_region | Meta-Analysis | COVID-19 positive vs COVID-19 negative | UKB | Imputed | Pan-ancestry | DOWN 0.962 [0.926, 0.999] | 0.04453 | Cases: 7771  (5627:  1229:  915) Controls: 35290 (25152:  6043:  4095) MAC:  17292 | 0.200784933 |

**Table S3: rs2048683 significantly associates with the list of studies extracted from the online platform (**[**https://rgc-covid19.regeneron.com/**](https://eur01.safelinks.protection.outlook.com/?url=https%3A%2F%2Frgc-covid19.regeneron.com%2F&data=05%7C01%7CNour.jalaleddine%40mbru.ac.ae%7Ce4fc00eecfb249f216dc08da22a76882%7Cc9a341c74b6f4e56b8d9f2325c282fda%7C0%7C0%7C637860399660061885%7CUnknown%7CTWFpbGZsb3d8eyJWIjoiMC4wLjAwMDAiLCJQIjoiV2luMzIiLCJBTiI6Ik1haWwiLCJXVCI6Mn0%3D%7C3000%7C%7C%7C&sdata=MfqVOOee7XZek9NIm2NHvHJevx8sp4eVR0H9d6X6JN0%3D&reserved=0)**)**

| **Gene** | **Variant** | **Variant type / HGVS** | **Analysis** | **Phenotype** | **Study** | **Genetic dataset** | **Ancestry** | **Effect (OR) [CI]** | **P Value** | **Case Counts / Controls (RR:RA:AA)** | **AAF** |
| --- | --- | --- | --- | --- | --- | --- | --- | --- | --- | --- | --- |
| **ACE2** | 23:15590376:  T:G | intronic | Single-Cohort | COVID-19 positive vs COVID-19 negative or COVID-19 status  unknown | UKB | Imputed | African (AFR) | DOWN 0.781 [0.659, 0.926] | 4.55E-03 | Cases: 227  (47:47:  133) Controls: 9193 (1240:  1965:  5988) MAC:  4586 | 0.7469 |
| **ACE2** | 23:15590376:  T:G | intronic | Single-Cohort | COVID-19 positive vs COVID-19 negative | UKB | Imputed | African (AFR) | DOWN 0.737 [0.588, 0.923] | 7.98E-03 | Cases: 227  (47:47:  133) Controls: 823 (90:196:537) MAC:  517 | 0.7447 |
| **ACE2** | 23:15590376:  T:G | intronic | Single-Cohort | COVID-19 positive vs COVID-19 negative or COVID-19 status unknown | UKB | Imputed | South Asian (SAS) | UP 1.22 [1.04, 1.42] | 1.33E-02 | Cases: 331  (41:53:  237) Controls:10252 (1676:  1804:  6772) MAC:  5291 | 0.7265 |
| **ACE2** | 23:15590376:  T:G | intronic | Single-Cohort | COVID-19 positive hospitalized vs COVID-19 negative or COVID-19 status unknown | GHS | Imputed | European (EUR) | UP 1.25 [1.04, 1.49] | 1.66E-02 | Cases: 180  (33:43:  104) Controls:112862 (25851:33515:  53496) MAC:  85326 | 0.6189 |
| **ACE2** | 23:15590376:  T:G | intronic | Single-Cohort | COVID-19 positive hospitalized vs COVID-19 negative or COVID-19 status unknown | UKB | Imputed | African (AFR) | DOWN 0.736 [0.569, 0.952] | 1.95E-02 | Cases: 83(21:  15:47) Controls: 9193 (1240:  1965:  5988) MAC:  4502 | 0.7477 |
| **N/A** | 23:15590376:  T:G | intergenic | Single-Cohort | Undefined | ANCESTRY | Imputed | European (EUR) | UP 1.17 [1.01, 1.35] | 3.32E-02 | Cases: -3  (-1:-1:-1) Controls: -3  (-1:-1:-1) MAC:  6329 | 0.6271 |
| **N/A** | 23:15590376:  T:G | intergenic | Single-Cohort | COVID-19 positive severe vs COVID-19 negative or COVID-19 status unknown | ANCESTRY | Imputed | European (EUR) | UP 1.16 [1, 1.33] | 4.49E-02 | Cases: -3  (-1:-1:-1) Controls: -3  (-1:-1:-1) MAC:  54875 | 0.6209 |
| **N/A** | 23:15590376:  T:G | intergenic | Meta-Analysis | Undefined | ANCESTRY | Imputed | Pan-ancestry | UP 1.14 [1, 1.31] | 4.65E-02 | Cases: -3  (-1:-1:-1) Controls: -3  (-1:-1:1) MAC:  7432 | 0.6561 |
| **N/A** | 23:15590376:  T:G | intergenic | Meta-Analysis | COVID-19 positive severe vs COVID-19 negative or COVID-19 status unknown | ANCESTRY | Imputed | Pan-ancestry | UP 1.14 [1, 1.29] | 4.87E-02 | Cases: -3  (-1:-1:-1) Controls: -3  (-1:-1:-1) MAC:  60811 | 0.6368 |

**Table S4**: **List of Human tagged primers used in the Fluidigm**

| **S No** | **Primer Name** | **Primer sequence** |
| --- | --- | --- |
| 1 | *ACE2*_1-1-F | ACACTGACGACATGGTTCTACAAGGGAAAGTCATTCAGTGGA |
| 2 | *ACE2*_2-2-F | ACACTGACGACATGGTTCTACATGTAACTGCTGCTCAGTCCA |
| 3 | *ACE2*_3-3-F | ACACTGACGACATGGTTCTACATGACCTTCAGCGGAGTAGAG |
| 4 | *ACE2*_4-4-F | ACACTGACGACATGGTTCTACAATAATGCTGGGGACAAATGG |
| 5 | *ACE2*_5-5-F | ACACTGACGACATGGTTCTACATCTCACAGTCAAGCTTCAGC |
| 6 | *ACE2*_6-6-F | ACACTGACGACATGGTTCTACAGGGCTATTCCTTCTCCACAT |
| 7 | *ACE2*_7_rs2285666-7-F | ACACTGACGACATGGTTCTACAGTTTGTAACCCAGATAATCC |
| 8 | *ACE2*_8-8-F | ACACTGACGACATGGTTCTACAGTGTGCTTTGGGATAACAGG |
| 9 | *ACE2*_9-9-F | ACACTGACGACATGGTTCTACACCAGCAAGGCTAATCTATGT |
| 10 | *ACE2*_10-10-F | ACACTGACGACATGGTTCTACAGGATTATTGGAGAGGAGACT |
| 11 | *ACE2*_11-11-F | ACACTGACGACATGGTTCTACATTCTAGTGTTGTGGAATGG |
| 12 | *ACE2*_12-12-F | ACACTGACGACATGGTTCTACAGCTGTGTTGTCATATACTA |
| 13 | *ACE2*_13-13-F | ACACTGACGACATGGTTCTACATCTGCTTCTACCAGTTCCAT |
| 14 | *ACE2*_14-14-F | ACACTGACGACATGGTTCTACACAAGGAGGCCGAGAAGTTC |
| 15 | *ACE2*_15-15-F | ACACTGACGACATGGTTCTACAGGGTACTCAAGATTCACTGG |
| 16 | *ACE2*_16-16-F | ACACTGACGACATGGTTCTACAATGTGCACAAAGGTGACAAT |
| 17 | *ACE2*_17-17-F | ACACTGACGACATGGTTCTACACCTACTCCAAATCCCTTAGC |
| 18 | *ACE2*_18-18-F | ACACTGACGACATGGTTCTACACCACTGTCATCTTCATCGTA |
| 19 | *ACE2*_19-19-F | ACACTGACGACATGGTTCTACAGCACTAGTTATGCCCACCT |
| 20 | *ACE2*_20-20-F | ACACTGACGACATGGTTCTACAGTAAGTAAACACGGGACTGC |
| 21 | *ACE2*_21-21-F | ACACTGACGACATGGTTCTACATGTGTAGCAATATGCTGAGG |
| 22 | *ACE2*_22-22-F | ACACTGACGACATGGTTCTACAGAGTCCCTCTGAGCAGTGT |
| 23 | *ACE2*_23-23-F | ACACTGACGACATGGTTCTACATTGTTTCTCTACAGGGAGGA |
| 24 | *ACE2*_24-24-F | ACACTGACGACATGGTTCTACAAAGTGTCCCCTTTGCTGTTT |
| 25 | *ACE2*_25-25-F | ACACTGACGACATGGTTCTACAGATACAGCCAACACTTGGAC |
| 26 | *ACE2*_26-26-F | ACACTGACGACATGGTTCTACAACACAGATTCCCCTGAAAC |
| 27 | *ACE2*_27-27-F | ACACTGACGACATGGTTCTACACCTTATGCCTCCATCGATAT |
| 28 | *ACE2*_28_ rs2106809-28-F | ACACTGACGACATGGTTCTACACTCACAGATCCCAAAACAGT |
| 29 | *ACE2*_28_ rs1978124-29-F | ACACTGACGACATGGTTCTACACTCACAGATCCCAAAACAGT |
| 30 | *ACE2*_29_rs2048683-30-F | ACACTGACGACATGGTTCTACACAGCTGTGTGATCTTGGACA |
| 31 | *ACE2*_30_rs4646188-31-F | ACACTGACGACATGGTTCTACAGTGTTCCCTTCTGTTGATGA |
| 32 | *ACE2*_32_rs879922-32-F | ACACTGACGACATGGTTCTACACCGTCATCAGCAATCAATAG |
| 33 | *ACE2*_33_rs4240157-33-F | ACACTGACGACATGGTTCTACAGGTGATTGTGGGACTATTTC |
| 34 | *ACE2*_34_rs4240157-34-F | ACACTGACGACATGGTTCTACAAGTCTCGGCAGATCAGGA |
| 35 | *ACE2*_35_rs233575-35-F | ACACTGACGACATGGTTCTACATCTCTCTGGTGGACTCTGAC |
| 36 | *ACE2*_36_rs233575-36-F | ACACTGACGACATGGTTCTACACCAGTGCTGAAGAAACTGG |
| 37 | *ACE2*_37_rs2074192-37-F | ACACTGACGACATGGTTCTACACAGAAGGGAAATTCTTGCC |
| 38 | *ACE2*_38-38-F | ACACTGACGACATGGTTCTACATATTCCACCCAACAGCTATG |
| 39 | *ACE2*_39-39-F | ACACTGACGACATGGTTCTACATAGAGAAGTGGAGGTGGATG |
| 40 | *ACE2*_40-40-F | ACACTGACGACATGGTTCTACACTCTTTGACAGTTCCCTTTG |
| 41 | ACE_41_rs879922-41-F | ACACTGACGACATGGTTCTACAGTCTTCTTGTTACTGGCAGT |
| 42 | *ACE2*_1-1-R | TACGGTAGCAGAGACTTGGTCTTAGAACAGGTCTTCGGCTTC |
| 43 | *ACE2*_2-2-R | TACGGTAGCAGAGACTTGGTCTTAGCCCAATAGAGCCATGAG |
| 44 | *ACE2*_3-3-R | TACGGTAGCAGAGACTTGGTCTGCAAGTGTGGACTGTTCCTT |
| 45 | *ACE2*_4-4-R | TACGGTAGCAGAGACTTGGTCTCCGTTTGCTCTTGTCTTCTG |
| 46 | *ACE2*_5-5-R | TACGGTAGCAGAGACTTGGTCTAGCTGCTGTGGGTGATATT |
| 47 | *ACE2*_6-6-R | TACGGTAGCAGAGACTTGGTCTGTACTGTAGATGGTGCTCATTG |
| 48 | *ACE2*_7_rs2285666-7-R | TACGGTAGCAGAGACTTGGTCTCTGCAGAGAAAATAAACCACTG |
| 49 | *ACE2*_8-8-R | TACGGTAGCAGAGACTTGGTCTGGCTTGGTAATGCAGAAGAA |
| 50 | *ACE2*_9-9-R | TACGGTAGCAGAGACTTGGTCTCGGCTGTAGTCATAGCCATC |
| 51 | *ACE2*_10-10-R | TACGGTAGCAGAGACTTGGTCTACTTTATGAGGCCTGGGAAT |
| 52 | *ACE2*_11-11-R | TACGGTAGCAGAGACTTGGTCTAGAGCCAAGTACACGAAGAA |
| 53 | *ACE2*_12-12-R | TACGGTAGCAGAGACTTGGTCTTAAGGGCTCTTTTTCCTACC |
| 54 | *ACE2*_13-13-R | TACGGTAGCAGAGACTTGGTCTCCCAGAATCCTTGAGTCATA |
| 55 | *ACE2*_14-14-R | TACGGTAGCAGAGACTTGGTCTCCTCTGTTGTCTCCCATTTA |
| 56 | *ACE2*_15-15-R | TACGGTAGCAGAGACTTGGTCTATGATGAGCTGTCAGGAAGT |
| 57 | *ACE2*_16-16-R | TACGGTAGCAGAGACTTGGTCTGCCATGAGAAAATGTCCATA |
| 58 | *ACE2*_17-17-R | TACGGTAGCAGAGACTTGGTCTCTTGTTTGAGCAGGAAGTTT |
| 59 | *ACE2*_18-18-R | TACGGTAGCAGAGACTTGGTCTCTTACTTCATCTCCCACCAC |
| 60 | *ACE2*_19-19-R | TACGGTAGCAGAGACTTGGTCTCTGTCCACAAACCCTAGAAG |
| 61 | *ACE2*_20-20-R | TACGGTAGCAGAGACTTGGTCTCCTAGGCATGGAAATGAGTA |
| 62 | *ACE2*_21-21-R | TACGGTAGCAGAGACTTGGTCTGAAACTTGTCAACTGGGTGT |
| 63 | *ACE2*_22-22-R | TACGGTAGCAGAGACTTGGTCTGAGAACCCAGCAAATCAAC |
| 64 | *ACE2*_23-23-R | TACGGTAGCAGAGACTTGGTCTCACACAGGAAGAACACACAA |
| 65 | *ACE2*_24-24-R | TACGGTAGCAGAGACTTGGTCTAATGCCAACCACTATCACTC |
| 66 | *ACE2*_25-25-R | TACGGTAGCAGAGACTTGGTCTAAGTCTAGGAAAGGCCACTT |
| 67 | *ACE2*_26-26-R | TACGGTAGCAGAGACTTGGTCTAAGGAGGTCTGAACATCATC |
| 68 | *ACE2*_27-27-R | TACGGTAGCAGAGACTTGGTCTTCAATGAAGATGCTCTCTCC |
| 69 | *ACE2*_28_ rs2106809-28-R | TACGGTAGCAGAGACTTGGTCTGTCAACCACACATACCACAA |
| 70 | *ACE2*_28_ rs1978124-29-R | TACGGTAGCAGAGACTTGGTCTGTCAACCACACATACCACAA |
| 71 | *ACE2*_29_rs2048683-30-R | TACGGTAGCAGAGACTTGGTCTAATGCTGCAGTGAACATGGC |
| 72 | *ACE2*_30_rs4646188-31-R | TACGGTAGCAGAGACTTGGTCTGGACTTTTCCCAGGAGTATG |
| 73 | *ACE2*_32_rs879922-32-R | TACGGTAGCAGAGACTTGGTCTATTCAAGGACTGGGGTTACT |
| 74 | *ACE2*_33_rs4240157-33-R | TACGGTAGCAGAGACTTGGTCTTACCAACCCTCACTGCAT |
| 75 | *ACE2*_34_rs4240157-34-R | TACGGTAGCAGAGACTTGGTCTCTGACCCTGTTGTGTAGATG |
| 76 | *ACE2*_35_rs233575-35-R | TACGGTAGCAGAGACTTGGTCTGTATTGGCATTTGGAGGTAG |
| 77 | *ACE2*_36_rs233575-36-R | TACGGTAGCAGAGACTTGGTCTTGTGACTGCTGTGTTTAAGG |
| 78 | *ACE2*_37_rs2074192-37-R | TACGGTAGCAGAGACTTGGTCTGCATCTTTCATGCCTTGC |
| 79 | *ACE2*_38-38-R | TACGGTAGCAGAGACTTGGTCTTTTGAGAGTTCCTCCTGGTA |
| 80 | *ACE2*_39-39-R | TACGGTAGCAGAGACTTGGTCTTAATGTGTGCCTCTGTGTGT |
| 81 | *ACE2*_40-40-R | TACGGTAGCAGAGACTTGGTCTGAGGCCATTGTAAACTGCT |
| 82 | ACE_41_rs879922-41-R | TACGGTAGCAGAGACTTGGTCTCCATCCTTATAACAGGTCGA |

*ACE2*: Angiotensin Converting Enzyme 2; F: Forward; R: Reverse
